# SOX2 promotes a cancer stem cell-like phenotype and local spreading in oral squamous cell carcinoma

**DOI:** 10.1371/journal.pone.0293475

**Published:** 2023-12-14

**Authors:** Alessandro Sacco, Anna Martina Battaglia, Gianluca Santamaria, Caterina Buffone, Selene Barone, Anna Procopio, Anna Maria Lavecchia, Ilenia Aversa, Emanuele Giorgio, Lavinia Petriaggi, Maria Giulia Cristofaro, Flavia Biamonte, Amerigo Giudice

**Affiliations:** 1 Department of Experimental and Clinical Medicine, Biochemistry and Molecular Biology Laboratory, "Magna Graecia" University of Catanzaro, Catanzaro, Italy; 2 Department of Health Sciences, School of Dentistry, “Magna Graecia” University of Catanzaro, Catanzaro, Italy; 3 Department of Experimental and Clinical Medicine, Biomechatronics Laboratory, “Magna Græcia” University of Catanzaro, Catanzaro, Italy; 4 Anatomical Pathology Unit, Pugliese-Ciaccio Hospital, Catanzaro, Italy; 5 Center of Interdepartmental Services (CIS), "Magna Graecia" University of Catanzaro, Catanzaro, Italy; Abu Dhabi University, UNITED ARAB EMIRATES

## Abstract

Emerging evidence shows that oral squamous cell carcinoma (OSCC) invasiveness can be attributed to a small subpopulation of cancer stem cells (CSCs) in the bulk of the tumor. However, the presence of CSCs in the OSCC close resection margins is still poorly unexplored. Here, we found that *BMI1*, *CD44*, *SOX2*, *OCT4*, *UBE2C*, *CXCR4* CSCs marker genes are significantly upregulated, while *IGF1-R*, *KLF4*, *ALDH1A1*, *CD133*, *FAM3C* are downregulated in the tumor core *vs* healthy mucosa of 24 patients with OSCC. Among these, *SOX2* appears also upregulated in the tumor close margin *vs* healthy mucosa and this significantly correlates with tumor size and lymph node compromise. *In vitro* analyses in CAL27 and SCC15 tongue squamous cell carcinoma cell lines, show that *SOX2* transient knockdown i) promotes the mesenchymal-to-epithelial transition, ii) smooths the invasiveness, iii) attenuates the 3D tumor sphere-forming capacity, and iv) partially increases the sensitivity to cisplatin treatment. Overall, our study highlights that the OSCC close margins can retain CSC-specific markers. Notably, *SOX2* may represent a useful CSCs marker to predict a more aggressive phenotype and a suitable target to prevent local invasiveness.

## Introduction

Oral squamous cell carcinoma (OSCC) accounts for more than 90% of head and neck cancers (HNSCC) [1,2]. Curative therapeutic approaches for OSCC include surgical resection, radiotherapy, and chemotherapy; however, according to the current clinical guidelines, surgery remains the conclusive treatment option for most patients [3–8]. One of the major concerns in the OSCC resection is sparing as much healthy tissue as possible to preserve vital functions and esthetics. At the same time, removing all malignant cells from the bulk of the tumor and around it is mandatory to reduce the possibility of local recurrence [3,8–11]. Although many efforts in defining “safe” surgical margins outside tumor boundaries have been achieved, local recurrence still affects up to 45% of OSCC patients [12–14]. In this regard, we and others have recently observed that OSCC close margins may retain molecular alterations that, otherwise, result undetectable through conventional histopathological examination, thus making the definition of “safe” tumor margins even more complex [11,15,16].

Local recurrence has been associated with two concepts: the minimal residual disease and the “field cancerization”. In the case of minimal residual disease, a small number of tumor cells, undetected by routine histopathology, remain in the margins upon surgery [17,18]. In the case of “field cancerization”, instead, a not macroscopically visible precancerous area surrounding the tumor stays behind unnoticed or can be detected as epithelial dysplasia [19,20]. Recently, the precancerous fields have been defined by the existence of genetic changes, either mutations, loss of heterozygosity (LOH), or copy number alterations (i.e., p53, CDKN2A, etc) [21–23]. However, the current detection methods of precancerous fields suffer from the problem of “undersampling”, as only a very small number of residual cancer cells are present in a relatively large tissue volume. Therefore, the identification of this small cell subpopulation, which likely represents the foci of precancerous initiation and progression, represents a major concern in oral cancer research.

The persistence of a very small fraction of cancer cells, defined as cancer stem cells (CSCs), provided with high tumorigenic proficiency, motility, invasion, and drug resistance, is largely considered a primary cause of tumor recurrence [24–27]. During the last decade, a few CSCs-related genes have been identified in OSCC lesions and associated, even often contradictorily, with nodal metastasis, chemoresistance, tumor recurrence, and survival rate of OSCC patients [25,28,29]. Among these, *SOX2* has been found overexpressed along the different stages of oral carcinogenesis, from potentially malignant oral disorders to invasive carcinomas [30]. SOX2, a member of the SOX family of high-mobility group transcriptional factors, holds a pivotal role in embryonic development and the preservation of stemness features in both embryonic and adult stem cell populations [29]. Robust evidence derived from preclinical studies involving both cell cultures and genetically modified mouse models strongly supports the role of SOX2 as an oncogene. SOX2, indeed, crosstalks with multiple signaling pathways to tightly regulate critical biological processes associated with tumor initiation and progression, such as cell-cycle, apoptosis, autophagy, and epithelial-to-mesenchymal transition (EMT). Dysregulation of SOX2 expression and activity occurs in several cancer types and often correlates with advanced tumor stages, unfavorable prognosis, and drug resistance [31].

The expression-based and the functional-based characterization of CSCs, as well as their spatial organization within the tumor mass and the surrounding tumor microenvironment (TME), is an attractive field of investigation as it may provide remarkable information on the existence of pre-metastatic niches.

In this study, we analyzed the expression-based distribution of CSCs within the bulk of the oral tumor and their close margin. To this, we analyzed the gene expression profile of CSCs markers and its potential impact on tumor recurrence and prognosis. Then, we assessed the functional role of putative CSCs markers in the OSCC cell phenotype *in vitro*.

## Results

### *SOX2* is overexpressed in the tumor core and its close margin of OSCC patients with lymph node compromise

According to previous findings of our [11] and other research groups (GEO databases, S1 Table) 13 CSCs markers genes (*BMI1*, *CD44*, *SOX2*, *OCT4*, *UBE2C*, *FAM3C*, *CXCR4*, *NANOG*, *RRM2*, *IGF-1R*, *KLF4*, *ALDH1A*, *CD133*) are differentially expressed in OSCC tissues compared to the relative healthy mucosa. Hence, to assess the distribution of tumor cells with CSCs-like phenotype in OSCC core and close margins, we performed the gene expression profile of these 13 CSCs markers in three different tissue samples (tumor core (T), tumor-free close margin (CM) and adjacent health distant margin (DM)) collected from a cohort of 24 patients with histologically confirmed OSCC. Clinicopathological, as well as demographic characteristics of the 24 patients enrolled in this study, are presented in Table 1.

**Table 1:** Demographic and clinicopathological characteristics of OSCC patients (n = 24).

VARIABLES			NUMBER (n)	PERCENTAGE (%)
Patient enrolled			24	
Age (years)	Mean (range)	68.6 (47 to 89)		
Sex	Male		14	58.3
	Female		10	41.6
Alcohol			11	45.8
Smoke			9	37.5
Site of tumor	Alveolar mucosa		6	25
	Tongue		15	62.5
	Cheek		3	12.5
Histologic grade	Well		5	20.8
	Moderate		16	66.6
	Poor		3	12.5
T stage	T1		14	58.3
	T2		4	16.6
	T3		5	20.8
	T4		1	4.1
N stage	N0		15	62.5
	N1		5	20.8
	N2		4	16.6
M stage	M0		24	100
	M1		0	0
TNM Stage	I		9	37.5
	II		3	12.5
	III		7	29.1
	IVA		5	20.8
Chemotherapy	No		18	75
	Yes		6	25
Radiotherapy	No		14	58.3
	Yes		10	41.6

**Fig 1:**
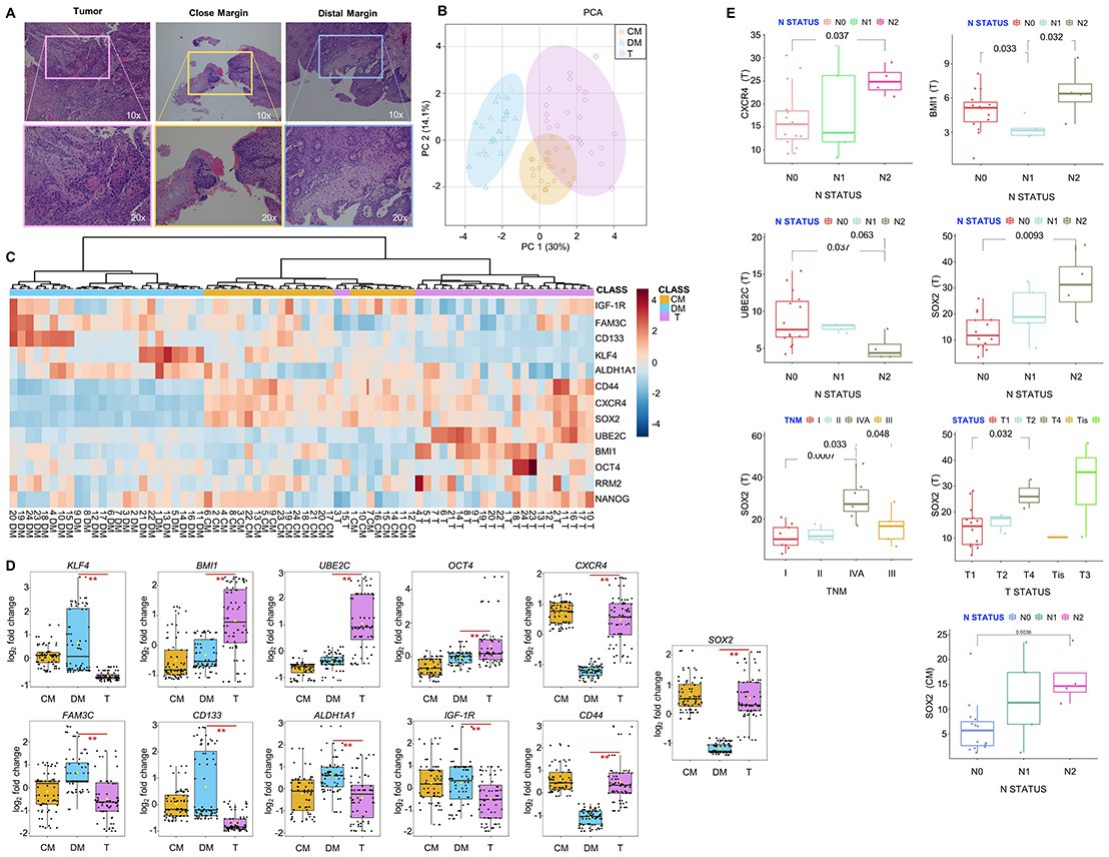
High *SOX2* in T and CM samples correlate with N status of patients with OSCC. **(A)** Representative images of tumor (T), close margin (CM), and distal margin (DM) tissue specimens stained with hematoxylin and eosin (10x and 20x magnification). **(B)** Principal component analysis (PCA), **(C)** unsupervised hierarchical clustering analysis, and **(D)** relative box plots showing gene expression pattern and levels (log_2_ |FC|) in T, CM, and DM samples (T *vs* DM, ** *p*-value < 0.01; CM *vs* DM, ** *p*-value < 0.01). **(E)** Correlation between *CXCR4*, *BMI*, *UBE2C*, *SOX2* expression in T and CM with TNM stage, tumor size (T status), and lymph node metastasis (N status) (*p*-value < 0.05 is statistically significant).

Briefly, the mean age of patients at recruitment was 68.6; the 58.3% was composed by men. Around half of patients routinely assumed alcohol (45.8%) and the 37.5% smoked. The main tumor site was the tongue (62.5%) with a preeminent moderate histological grade (66.6%). TNM stage was well distributed among the four categories. The main therapeutic approach was radiotherapy, while chemotherapy was used only in the 25% of patients. Only primary OSCC samples with histologically tumor-free CM were included in the study. Indeed, as shown in the representative images of hematoxylin and eosin staining, T showed neoplastic proliferation of pleomorphic cells, CM showed a mild dysplasia with atypical tissue morphology only at the inner epithelial layers, and DM showed a mild hyperplasia without any signs of disrupted tissue morphology (10x and 20x images in Fig 1A). First, Principal Component Analysis (PCA) and unsupervised hierarchical clustering analysis confirmed that, overall, T samples were consistently different from DM samples (Fig 1B and 1C). In detail, *SOX2*, *BMI1*, *UBE2C*, *OCT4*, *CXCR4*, and *CD44* were significantly upregulated in T *vs* DM (Log_2_|FC| >1, ** *p*-value < 0.01) while *KLF4*, *FAM3C*, *ALDH1A1*, *CD133*, and *IGF-1R* were significantly down-regulated in T *vs* DM (Log_2_|FC| <1, ** *p*-value < 0.01) (Fig 1D). Then, we interestingly found that CM samples appeared partially clustering with T (Fig 1B and 1C). Indeed, for some patients, *SOX2*, *CXCR4*, and *CD44* expression levels in CM samples were similar to those observed in their relative T samples and higher than those observed in DM (Fig 1D). For *SOX2*, this trend was confirmed also at the protein level by Western Blot (WB) (see patient #2 in S1 Fig). No significant differences were observed in *NANOG* and *RRM2* (S2 Fig).

Analysis of clinicopathological correlations showed that high levels of *SOX2* in T significantly correlated with a higher TNM stage [32] (stage IVA vs stage I, *p*-value = 0.0007; stage IVA vs stage II, *p*-value = 0.033; stage IVA vs stage III, *p*-value = 0.048) while high levels of *CXCR4* and *BMI1* significantly correlated with a greater lymph node compromise (N status) (*CXCR4*: N2 vs N0, *p*-value = 0.037) (*BMI1*: N2 vs N1, *p*-value = 0.032). Patients with greater N status also showed significantly lower *UBE2C* levels in T (N2 vs N0, *p*-value = 0.037). Notably, high levels of *SOX2* in CM significantly correlated with lymph node compromise (N2 *vs* N0, log_2_ |FC| = 1.32, *p*-value = 0.0036). Overall, these data suggest that SOX2 might be involved in OSCC local spreading.[NO_PRINTED_FORM]

### TCGA analysis of CSCs markers in HNSCC

**Fig 2:**
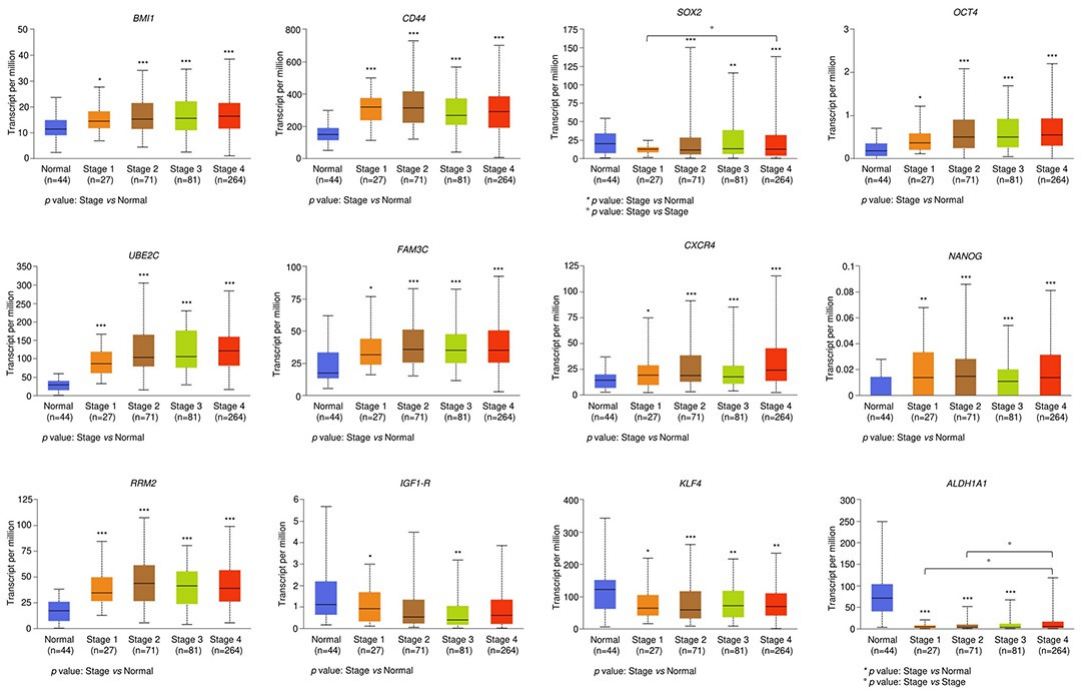
Correlation between CSCs markers and tumor stage in HNSCC patients. Correlation between the expression of CSCs markers and tumor stage in a cohort of primary HNSCC tissue specimens (n = 520) derived from TCGA dataset. CSCs expression markers are reported also in healthy mucosa samples (normal, n = 44). *p-*value Stage *vs* Normal: * < 0.05, ** < 0.01, *** < 0.001; *p*-value Stage *vs* Stage: ° < 0.05.

**Fig 3:**
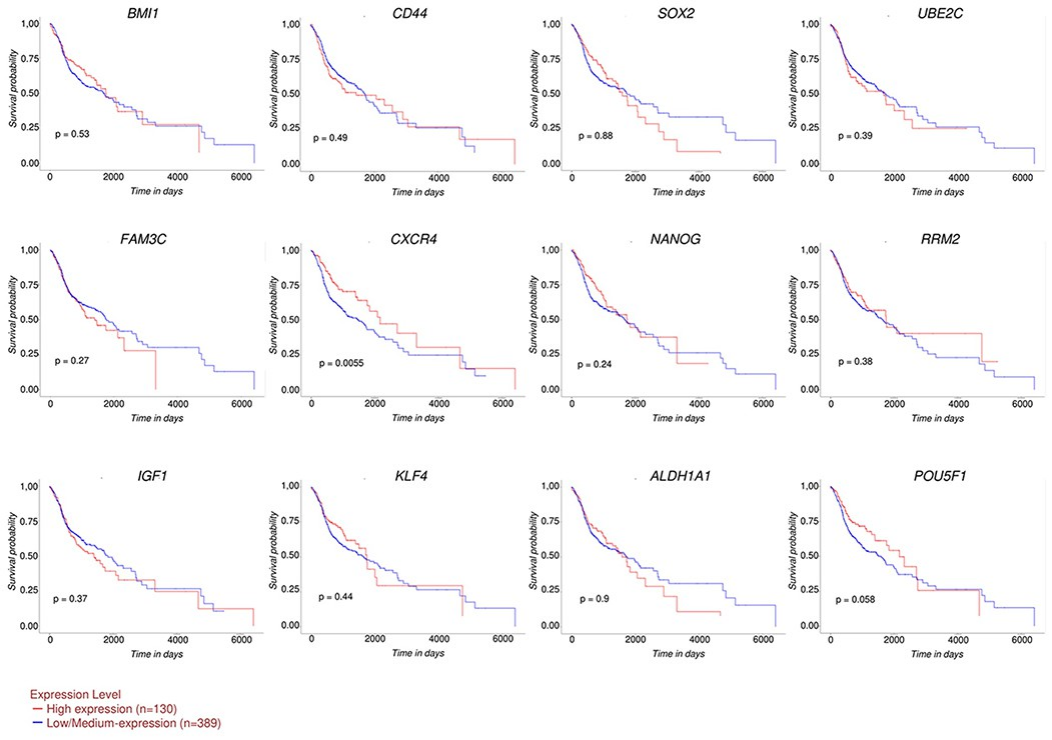
Impact of CSCs markers expression on overall survival (OS) of HNSCC patients. Kaplan-Maier OS curves of patients harboring high (n = 130, red line) and low/medium (n = 389, blue line) expression of CSCs markers. *p*-value < 0.05 was considered statistically significant.

OSCC accounts for more than 90% of HNSCC [1,2]. Thus, we employed the RNA-seq data relative to 520 primary HNSCC tissue specimens and 44 healthy mucosa samples archived in TCGA to confirm the impact of the above mentioned 13 CSCs markers on patient outcomes. We found that *BMI1*, *CD44*, *SOX2*, *OCT4*, *UBE2C*, *FAM3C*, *CXCR4*, *NANOG*, and *RRM2* mRNA levels were significantly increased while *IGF-1R*, *KLF4*, and *ALDH1A* were decreased in OSCC samples compared to healthy adjacent mucosa and that this trend significantly correlated with tumor stage (Fig 2). Patients harboring low/medium expression of *CXCR4* in tumor samples showed a worse overall survival (OS) (*p*-value = 0.0055). No significant impact on OS was observed for all the other DEGs (Fig 3).

### *SOX2* knockdown reduces OSCC cell migration ability

**Fig 4:**
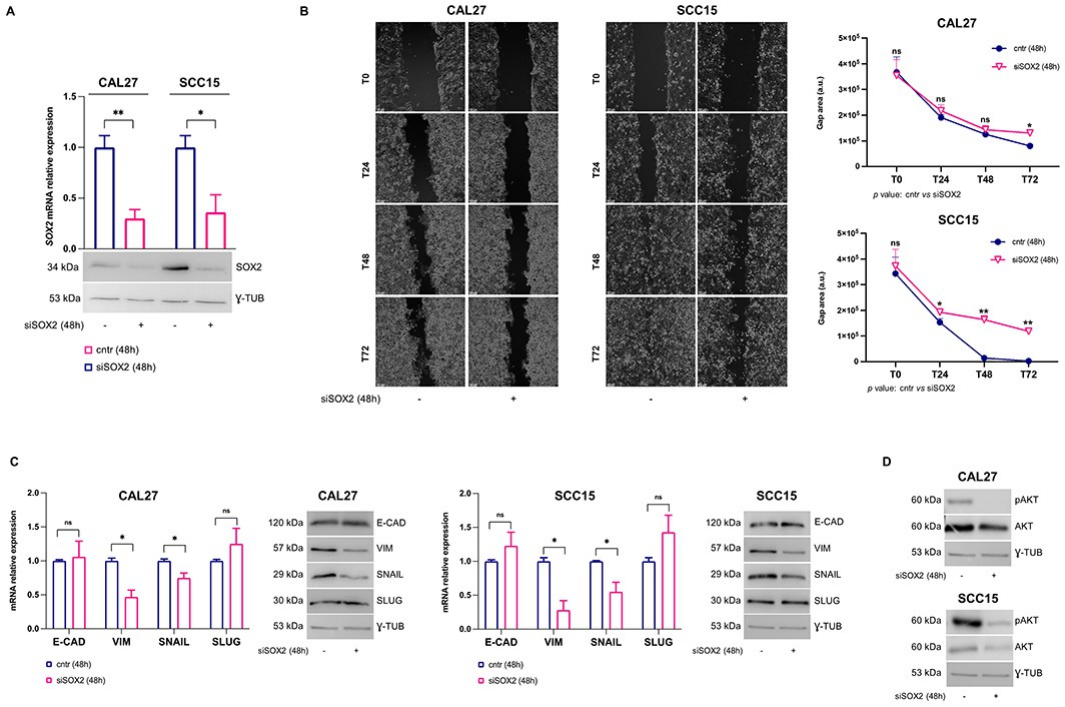
*SOX2* knockdown decreases OSCC cell migration and EMT. **(A)** qRT-PCR and western blot analyses of SOX2 in CAL27 and SCC15 cells (siSOX2 *vs* cntr). γ-TUB was used as a normalization control for protein quantification. **(B)** Representative images of wound healing assay of CAL27 and SCC15 cells (siSOX2 *vs* cntr) at T0, T24, T48, and T72 (10x magnification) (left); Mean and SD of the gap area (a.u.: arbitrary unit) of three biological replicates (right). **(C)** qRT-PCR and WB analysis of EMT markers in both cell lines upon *SOX2* knockdown. **(D)** WB analysis of pAKT/AKT in both cell lines upon *SOX2* knockdown. All the experiments were carried out in triplicate. qRT-PCR are presented as mean ± SD. *p-*value: *< 0.05, ** < 0.01. ns: not significant.

To investigate the contribution of SOX2 in the development of an aggressive and invasive OSCC phenotype, we performed its transient knockdown in CAL27 and SCC15 OSCC cell lines (Fig 4A). Then, we performed wound healing assays upon 24h, 48h and 72h transfection with siSOX2 and, as reported in representative images and relative histograms, we observed that *SOX2* knockdown reduced *in vitro* migration of both CAL27^siSOX2^ and SCC15^siSOX2^ (*p-value < 0.05, 72h CAL27) (*p-value < 0.05, 24h SCC15) (**p-value < 0.01, 48h SCC15) (**p-value < 0.01, 72h SCC15) (Fig 4B; S1–S4 Movies). Because tumor migration is tightly associated with epithelial-to-mesenchymal transition (EMT), we further measured the expression levels of key EMT markers vimentin (*VIM*) and e-cadherin (*E-CAD*) and mediators (*SNAIL* and *SLUG*). Interestingly, we found that *SOX2* knockdown strongly reduced both mRNA and protein expression of the mesenchymal marker *VIM* and that of the EMT-associated transcription factor *SNAIL* in both cell lines (*p-value <0.05) (Fig 4C). In agreement with *in vitro* data, we found that both in T and CM samples, higher levels of *SOX2* correlate with lower levels of *E-CAD* and higher levels of *VIM*, compared to T and CM samples showing low levels of *SOX2* (*p-value < 0.05) (S3A and S3B Fig). Finally, WB analyses showed that SOX2 knockdown was associated with the reduction of pAKT protein levels thus suggesting a repression of AKT signaling pathway (Fig 4D).

### *SOX2* knockdown attenuates CSCs-associated properties of OSCC cells

**Fig 5:**
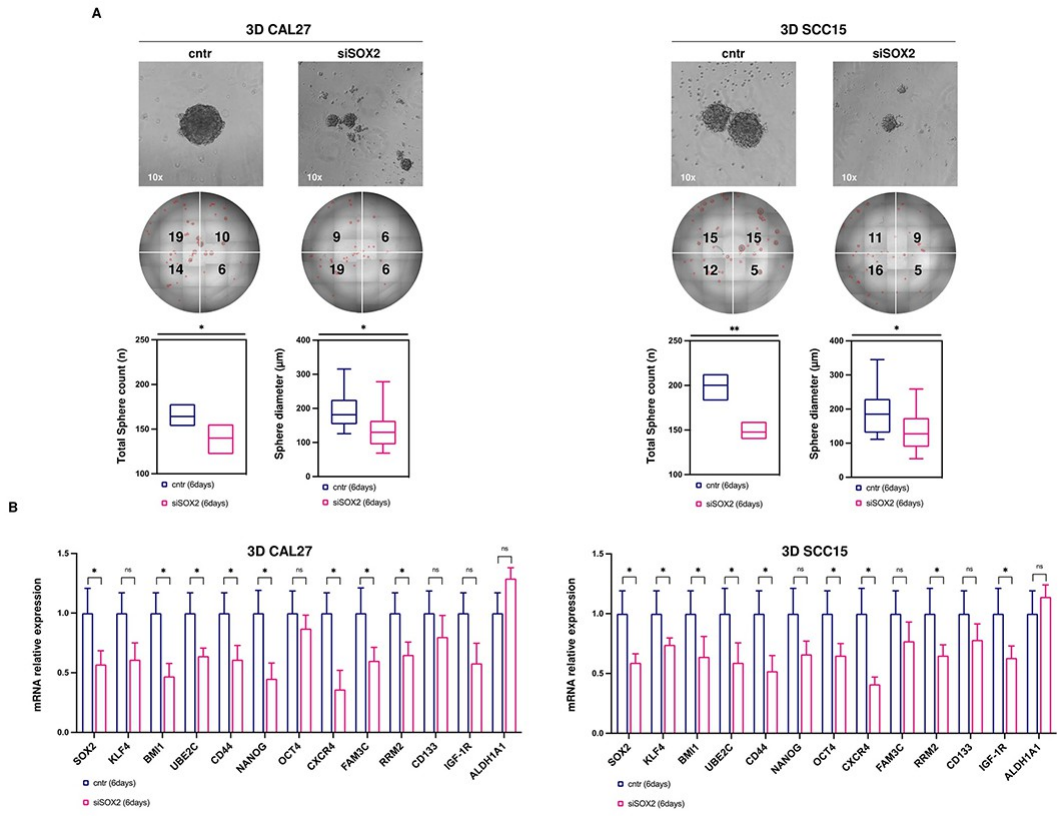
*SOX2* knockdown mitigates the stemness properties of OSCC cells. **(A)** Representative images and relative histograms of 3D tumor spheroid morphology, count, and diameter in CAL27 and SCC15 cells (siSOX2 *vs* cntr). **(B)** qRT-PCR analysis of a panel of stemness genes in both cell lines upon *SOX2* knockdown. All the experiments were carried out in triplicate and results are presented as mean ± SD. *p-*value: *< 0.05, ** < 0.01. ns: not significant.

Next, we tested the effect of *SOX2* silencing on OSCC cell anchorage-independent growth by performing 3D tumor spheroid assay. As reported in representative images and relative histograms, *SOX2* knockdown significantly reduced the number of tumor spheroids from a mean of 164.21 to 139.81 in CAL27 and from 200.15 to 147.65 in SCC15 cells (*p-value < 0.05, CAL27) (**p-value < 0.01, SCC15). Moreover, *SOX2* knockdown significantly also affected the spheroids diameter as demonstrated by the reduction from 193.66 μm to 134.39 μm in CAL27 and from 192.39 μm to 131.26 μm in SCC15 cells (*p-value < 0.05) (Fig 5A; S5–S8 Movies). In line with these results, we found that *SOX2* knockdown decreased the expression of a consistent number of other CSCs markers (*BMI1*, *UBE2C*, *CD44*, *NANOG*, *CXCR4*, *FAM3C*, and *RRM2*) in both CAL27 and SCC15 3D tumor spheroids (*p-value <0.05) (Fig 5B).

### Targeting *SOX2* improves sensitivity to cisplatin treatment in OSCC cells

**Fig 6:**
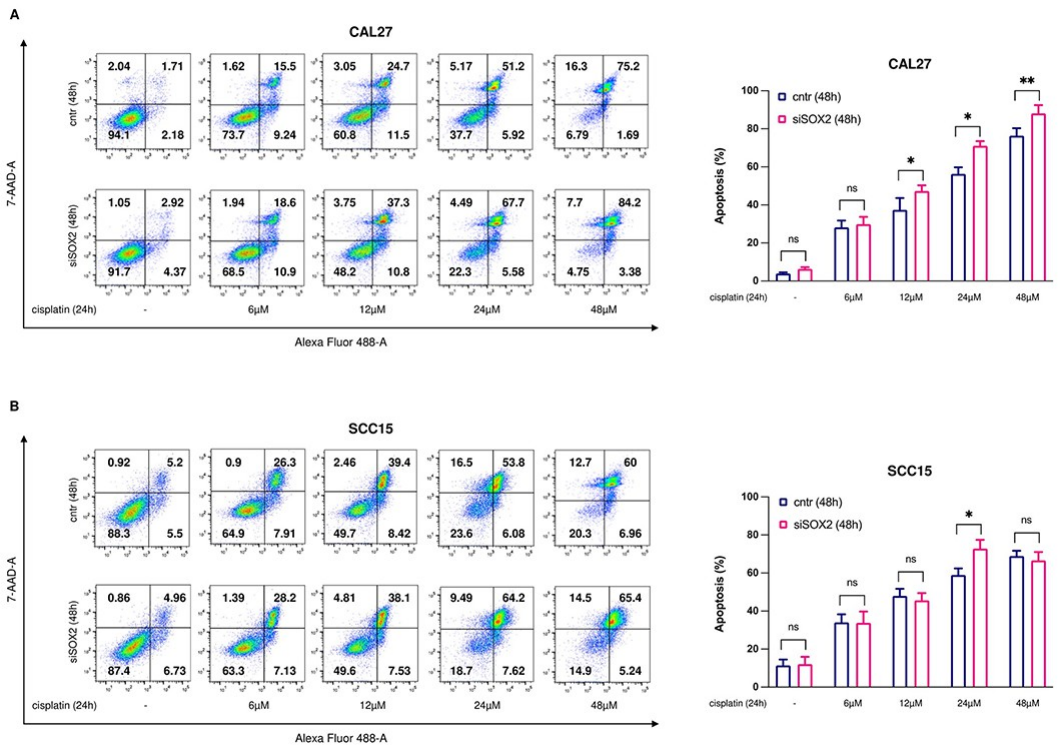
*SOX2* silencing increases apoptosis in OSCC cells upon cisplatin administration. **(A-B)** Representative plots of Annexin V/7-AAD apoptosis assay (left) in CAL27 and SCC15 cells (siSOX2 *vs* cntr) upon treatment with 6, 12, 24 and 48μM cisplatin (24h) and relative graphical data of total apoptotic cells (%) (right). All the experiments were carried out in triplicate and results are presented as mean ± SD. *p-*value: *< 0.05, ** < 0.01. ns: not significant.

The observation that *SOX2* supports oral squamous CSCs properties was suggestive of its possible involvement in the modulation of response of OSCC to chemotherapy. Thus, we treated CAL27^siSOX2^ and SCC15^siSOX2^ cells and relative control cells with growing concentration of cisplatin (6μM, 12μM, 24μM, 48μM) for 24h upon 48h transient transfection with siSOX2 and negative control siRNA, respectively. As shown in Fig 6A and 6B, *SOX2* knockdown significantly improved sensitivity of CAL27 cells at 12μM, 24μM and 48μM of cisplatin (*p-value <0.05 12μM and 24μM) (**p-value <0.01 48μM) by increasing the percentage of cells undergoing apoptosis compared to control cells. This effect was confirmed in SCC15 cells only upon treatment with 24μM cisplatin (*p-value <0.05).

## Discussion

It is well known that the OSCC core is populated by a small fraction of CSCs, which may represent the culprits behind the anchorage-independent growth, migration, and spreading of tumor cells [33]. The presence within the OSCC margins of a subpopulation of CSCs that may function as cancer-initiating cells and, thus. may massively contribute to local recurrence, still remains obscure because of the lack of surrogate biomarkers.

**Fig 7:**
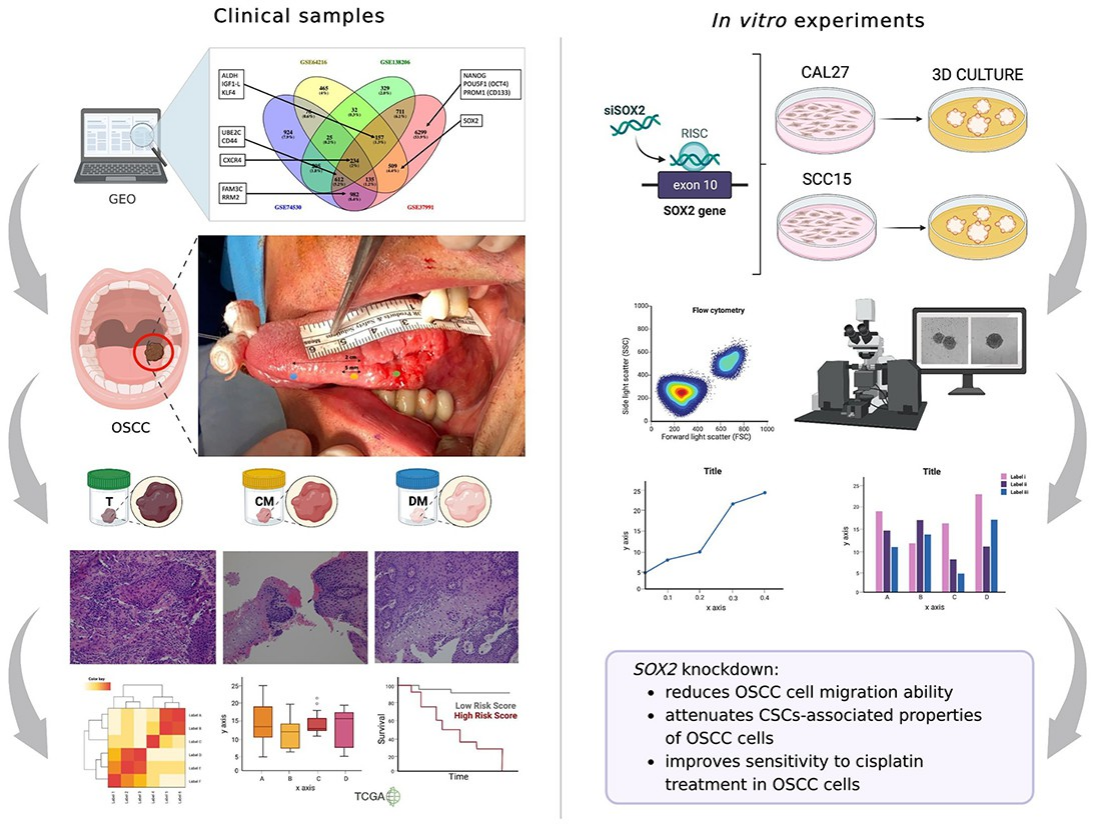
Schematic representation of the investigation workflow.

In the present study, whose experimental workflow is schematically reported in Fig 7, we demonstrate that the OSCC is defined by a CSCs-associated molecular signature characterized by high levels of *SOX2*, *BMI1*, *UBE2C*, *OCT4*, *CXCR4*, and *CD44* and low levels of *CD133*, *KLF4*, *ALDH1A1*, and *IGF-1R*. Notably, high levels of *SOX2*, *BMI1*, and *CXCR4*, as well as low levels of *UBE2C* in the tumor core (T) significantly correlate with a greater tumor size and lymph node compromise. Due to the short follow-up period (<3 years), our cohort of patients did not show distant metastasis (M status) or death events, so correlations with M status or OS were not available. These results were supported by TCGA analysis showing that the identified CSCs gene signature significantly correlates with advanced tumor stage, but not with OS, also in patients with HNSCC. [24,27,28,33–41]. The only exception was for *CXCR4*, whose high expression in HNSCC samples appears to correlate with a better OS.

The main finding of our study is that the close margin, defined as tumor-free by the conventional histopathological examination, may show high levels of *SOX2*, *CD44*, and *CXCR4* to an extent comparable to that observed in the relative tumor core. Most importantly, the high expression of *SOX2* in the close margin significantly correlates with a greater lymph node compromise, thus suggesting a possible role of this marker in fostering the progression of OSCC. Our results add an important piece of information to the current literature that, although seems to agree on the overexpression of SOX2 along the different steps of oral cancer [30], still remains debated on the role of SOX2 in OSCC progression [29,31,30] SOX2 has been correlated either to a favorable or an unfavorable prognosis. In support of a role as favorable prognostic biomarker, it has been reported that *SOX2* is overexpressed during the early stages of OSCC and that this predicts for reduced local recurrence [42]. On the contrary, other studies have demonstrated that SOX2 overexpression promotes EMT and lymph node metastasis [42–47] while SOX2 knockdown inhibits HNSCC cell self-renewal and chemoresistance [34]. A recent retrospective study also highlights that *SOX2* is overexpressed in pre-malignant oral disorders and that this predicts for oral cancer evolution [42, 48].

A survey of the literature indicates that SOX2 affects tumor progression by acting on several signaling pathways in a context-dependent manner. SOX2 may promote EMT by transcriptionally activating SNAIL, SLUG, and TWIST, which in turn act as transcription factors involved in the repression of the epithelial marker E-CADHERIN and the activation of the mesenchymal marker VIMENTIN. In breast and pancreatic cancer, indeed, the overexpression of SOX2 leads to the EMT through the repression of the epithelial genes *E-CAD* and *Zo-1* [49,50]. SOX2 may promote the remodeling of the extracellular matrix (ECM). In lung cancer, indeed, SOX2 activates AKT/mTOR signaling pathway, which in turn enhances the activity of the matrix-metalloproteinase-2 (MMP2) [51]. [49–51] In prostate cancer, SOX2 fosters the EMT by directly binding to the β-catenin enhancer and thus activating the Wnt/β-catenin pathway [52]. In this regard, in this study we show that *SOX2* knockdown remarkably reduces the OSCC cells migration ability and that is associated with inhibition of the EMT as suggested by the significant reduction of SNAIL and VIMENTIN and the slight increase of E-CADHERIN. Furthermore, we show that *SOX2* silencing is associated with the repression of AKT signaling pathway as demonstrated by the reduction of pAKT protein levels.

In bladder cancer, tumor progression is associated with the strong up-regulation of SOX2 and NANOG and the consequent acquisition of cancer stem cell properties [53,54]. According to our results, silencing *SOX2* leads to the inhibition of anchorage-independent growth of CAL27 and SCC15 cells and, thus, to the significant reduction of 3D oral tumor spheroids generation.

A previous study highlighted that, in HNSCC, SOX2 triggers cisplatin resistance by promoting drug efflux via activation of the ATP-binding cassette ABCG22 (ABCG2) transporter [55]. In ovarian cancer SOX2 ectopic overexpression promotes tumor cell resistance to several chemotherapeutic compounds, such as cisplatin, carboplatin, and paclitaxel, by altering the homeostasis between pro- and anti-apoptotic proteins. Conversely, *SOX2* knockdown restores drug sensitivity by causing mitochondrial dysfunction, apoptosis, and autophagy [56]. In EGFR-mutated lung cancer cells, *SOX2* overexpression promotes resistance to erlotinib by repressing the expression of proapoptotic proteins BIM and BMF [57]. Concerning the role of SOX2 in OSCC response to chemotherapy, our results indicate that the loss of *SOX2* improves CAL27 and SCC15 sensitivity to cisplatin treatment by increasing the percentage of apoptotic events. However, further detailed mechanisms underlying the contribution of *SOX2* to a more aggressive behavior remain to be entirely elucidated and will be subject of future studies.

Collectively, our study underlines that the molecular analysis highlights the existence of residual tumor cells over-expressing CSCs markers even in the OSCC close margins defined as tumor-free by the conventional histopathological examination. In particular, we show for the first time that *SOX2* is over-expressed not only in the tumor core but also in the close margin of OSCC patients with advanced tumor stage and lymph node compromise, thus suggesting that high levels of *SOX2* may predict for a local spreading. Furthermore, we demonstrate that *SOX2* knockdown abrogates *in vitro* OSCC cell migration ability and CSCs-traits, and at the same time, improves OSCC cell sensitivity to cisplatin. Future *in vitro* and *in vivo* studies are mandatory to investigate the molecular mechanisms underlying the role of *SOX2* in OSCC spreading and its potential as therapeutic target to prevent OSCC dissemination.

## Materials and methods

### In silico analysis through Gene Expression Omnibus (GEO database)

CSCs markers genes expression were explored using GEO database. Research queries included “Homo sapiens” and “oral squamous cell carcinoma” as keywords. After a survey of the literature, 4 datasets containing CSCs gene expression profiles of both OSCC, and healthy oral tissues were selected (S1 Table) and analyzed in R Software (Version: 1.3.1093) by using “limma” bioconductor package to identify the differentially expressed genes (DEGs) between OSCC and healthy tumor tissues. DEGs were selected by using the following criteria:log_2_|FC| ≥1 and adjust *p*-value ≤ 0.05.

### Patients and clinical samples

Twenty-four OSCC patients were surgically treated at the Oral Pathology and Oral Surgery Unit of “Magna Graecia” University, between December 2020 and December 2022. For each patient, primary tissue specimens were collected at 2 cm from the macroscopic lesion boundaries defined visually and by palpation. Laser Capture Microdissection (LCM) was used to obtain three different area from each biopsy: i) tumor core (T), ii) tumor-free close margin (CM) within 1–4.9 mm from the lesion boundaries and iii) adjacent health distant margin (DM) collected > 1.5 cm from the lesion boundaries, as recommended from current clinical guidelines [7,10,58,59]. Two μm serial sections were obtained from formalin-fixed and paraffin-embedded T, CM and DM tissue specimens. Samples collection was performed in the same two-year’s time frame.

All patients provided a written informed consent at the time of data collection.

No information that could identify individual participants are available. The procedures reported in this study were performed in accordance with the Helsinki Declaration guidelines (2008) on human experimentation and good clinical practice (good clinical practice or GCP).

### TCGA data analysis

A total of 564 samples were analyzed by TCGA Biolinks R Bioconductor [60] for the expression of 13 selected CSCs marker genes. OSCC samples were selected using ALCAN database, (http://ualcan.path.uab.edu/cgi-bin/ualcan-res.pl) [61].

### Cell lines and culture

OSCC cell lines CRL-2095 (CAL27) and CRL-1623 (SCC15) derived from tongue squamous carcinoma were purchased from the American Type Culture Collection (ATCC, Rockville, MD, USA). CAL27 cells were grown in Dulbecco’s Modified Eagle’s Medium (DMEM) (Sigma-Aldrich, St. Louis, MO, USA) supplemented with 10% (v/v) Fetal Bovine Serum (FBS) (Invitrogen, San Diego, CA, USA), and 1% (v/v) of penicillin and streptomycin 100 U/ml (Sigma-Aldrich, St. Louis, MO, USA). Conversely, for SCC15 cell line a 1:1 mixture of DMEM medium (Sigma-Aldrich, St. Louis, MO, USA) and Ham’s F-12 Nutrient Mix (Thermo Fisher Scientific, Waltham, MA, USA), supplemented with 90% (v/v) hydrocortisone 400 ng/ml (Sigma-Aldrich, St. Louis, MO, USA), 10% FBS, and 1% (v/v) of penicillin/streptomycin 100 U/ml was used. Cells were maintained in a 5% CO_2_ humidified atmosphere at 37°C and routinely examined for Mycoplasma contamination. CAL27 and SCC15 were selected for their ability to generate three-dimensional (3D) tumor spheroid *in vitro*.

### RNA isolation, cDNA generation, and Real-time quantitative reverse transcription (qRT)-PCR

Total RNA was isolated using TRIzol™ Reagent (Life Technologies, Carlsbad, CA, USA) according to the manufacturer’s instructions. Extracted RNA quality and quantification were assessed using NanoDrop® ND-1000 (Thermo Fisher Scientific, Waltham, MA, USA) [62–64]. Then, 1μg of total RNA were retrotranscribed using High-Capacity cDNA Reverse Transcription Kit (Thermo Fisher Scientific, Waltham, Massachusetts, USA). Synthesized cDNA (50 ng) was used for qRT-PCR performed using SYBR™ Green PCR Master Mix (Thermo Fisher Scientific, Waltham, Massachusetts, USA), and 400 nM of each primer pair [65]. Genes analyzed were as follow: *E-CAD*, *VIM*, *SNAIL*, *SLUG*, *SOX2*, *KLF4*, *BMI1*, *UBE2C*, *CD44*, *NANOG*, *OCT4*, *CXCR4*, *FAM3C*, *RRM2*, *CD133*, *IGF-1R*, and *ALDH1A1*. The thermal profile used were structured as follow: 1 step at 95°C for 10 min, 45 cycles at 95°C for 30 sec, and 60 sec at 60°C. The relative mRNA expression level was calculated by the 2^−ΔΔCt^ method using *GAPDH* or *RPL38* as housekeeping genes for cell lines or tissue specimens, respectively [66]. Each reaction was performed in triplicate.

### Western blot

Protein extraction and Western Blot were performed as previously reported by Chirillo R et al [67]. Antibodies against SOX2 (1:500, sc-365823), VIM (1:500, sc-7557), E-CAD (1:500, sc-8426), SLUG (1:500, sc-166476), SNAIL (1:500, sc-393172) were purchased from Santa Cruz Biotechnology (Santa Cruz Biotechnology, Dallas, Texas, USA); antibodies against AKT (1:1000, #9272) and p-AKT (1:1000, #9271) were obtained from Cell Signaling Technology (Danvers, Massachusetts, USA). Goat polyclonal anti-γ-Tubulin antibody (1:3000; sc-7396) (Santa Cruz Biotechnology, Dallas, TX, USA) and RPL38 (1:1000, PA5-88313) (Thermo Fisher, Waltham, MA, USA) served as a reference for samples loading. The membranes were washed for 30 min with T-TBS solution and then incubated for 1h at room temperature with peroxidase-conjugated secondary antibodies (Peroxidase AffiniPure Sheep Anti-Mouse IgG, 1:10000; Peroxidase AffiniPure Donkey Anti-Goat IgG, 1:10000) (Jackson ImmunoResearch Labs, West Grove, PA, USA). Chemiluminescence signals were detected using Western Blotting Luminol Reagent (Santa Cruz Biotechnology, Dallas, TX, USA) and acquired by UVItec Alliance Mini HD9 (UVItec Ltd. Cambridge, Cambridge, UK). The protein band intensity on western blots was quantified and normalized using ImageJ Software (http://rsb.info.nih.gov/ij/) [68–70].

### *SOX2* transient knockdown

CAL27 and SCC15 cells were transfected using Lipofectamine™ 3000 Transfection Reagent (Thermo Fisher Scientific, Waltham, MA, USA) according to the manufacturer’s protocol. SOX2 siRNA was purchased from Thermo Fisher Scientific. To ensure an optimal control, the two cell lines were further transfected with Silencer™ Select Negative Control siRNA (cntr) (Thermo Fisher Scientific, Waltham, MA, USA). The evaluation of transfection efficiency was performed by western blot and qRT-PCR at 48h [71].

### Wound healing assay

1x10^6^ cells/well were seeded in 6-wells standard plates. To simulate a wound, the cells monolayer was manually scratched using a pipette tip. Wound closure was then monitored through images and time-lapse video recorded at 0, 24 and 48h using Leica THUNDER Imaging Systems DMi8 (Leica Microsystems S.r.l., Wetzlar, Germany). Subsequently, cell migration was quantified by Leica Application Suite Software [72].

### 3D tumor spheroid assay

CAL27 and SCC15 were seeded in Corning® Costar® Ultra-Low Attachment Multiple Well Plates (Corning Inc., New York, NYC, USA) at a concentration of 15x10^3^ cells/mL. OSCC 3D tumor spheroids were cultured in a previously described sphere medium [73]. OSCC tumor spheroids were grown in a 5% CO_2_ humidified atmosphere at 37°C and monitored through images and time-lapse video recorded for 6 days using Leica THUNDER Imaging Systems DMi8 (Leica Microsystems S.r.l., Wetzlar, Germany). The collected tumor spheroids were resuspended in an appropriate volume of culture medium and counted according to the following formulas:

sphereconcentration=spherecount÷countingvolume(μL)

totalspherecount=sphereconcentration×totalvolume

Their diameters were then measured through the internal image measuring feature of Leica Application Suite Software.

### Cisplatin treatment

CAL27^cntr^, CAL27^siSOX2^, SCC15^cntr^, SCC15^siSOX2^ were seeded in 6-wells standard plates. Cisplatin was added into the medium at various concentrations (6μM, 12μM, 24μM, 48μM) for 24h. Treatment was performed upon 48h of transient transfection with siSOX2 or negative control siRNA, respectively.

### Flow cytometry apoptosis analysis

To identify cells actively undergoing apoptosis, a double staining with Annexin V and PI was performed using Alexa Fluor®488 Annexin V/Dead Cell Apoptosis Kit (Thermo Fisher Scientific, Waltham, MA, USA) according to the manufacturer’s instructions. Cells were then incubated at room temperature for 15 min in the dark. Each tube was diluted with 400μL of Annexin Binding Buffer. Flow cytometry assays were performed using the BD LSRFortessa™ X-20 (BD Biosciences, San Jose, CA, USA). Data analysis was carried out using FlowJo™ v10 Software (BD Biosciences, San Jose, CA) [72,74,75].

### Statistical analysis

Statistical tests were conducted using GraphPad Prism 9 and R software. Gene expression data were analyzed by Principal Component Analyisis (PCA) and unsupervised hierarchical clustering. ANOVA multi-sample test (permutation-based 5% FDR) was performed on the resulting dataset, and the significant DEGs were grouped by unsupervised hierarchical clustering. Multivariate correlations between gene expression data and clinicopathological parameters were performed using a Cox’s multiple linear regression model based on Firth’s bias correction method. The statistical significance of the *in vitro* experimental data was analyzed using the two-tailed Student’s t-test (for comparisons of two treatment groups) or one-way ANOVA (for comparisons of three or more groups). All results were expressed as the means ± standard deviation (SD). A *p-*value ≤ 0.05 was considered statistically significant.

## Supporting information

S1 Fig SOX2 protein levels in T, CM, and DM of two representative OSCC samples. WB analysis of SOX2 in T, CM, and DM samples of two representative OSCC patients (#1 and #2) and relative optical densitometry. In patient #1, SOX2 protein level is higher in T compared to CM and DM. In patient #2, SOX2 protein level is similar in T and CM and higher compared to DM. RPL38 was used as a normalization control for protein quantification.
(PDF)

S2 Fig *NANOG* and *RRM2* did not show any significant difference among T, CM and DM samples. Box plots showing *NANOG* and *RRM2* gene expression levels (log_2_ |FC|) in T, CM, and DM samples (ns: not significant).
(PDF)

S3 Fig High *SOX2* levels correlate with EMT phenotype in tissue samples. Box plots showing the relative expression of *VIM* and *E-CAD* in both T **(A)** and CM **(B)** samples clustered according to high or low *SOX2* levels, respectively (*ANOVA t-test *p* value < 0.05).
(PDF)

S1 Table Details of the selected four databases. (PDF)

S1 Raw images Uncropped plots relative to all Western Blot analyses. **(A)** Uncropped plot relative to Western Blot analysis (showed in Fig 4A) of SOX2 in CAL27 and SCC15 cells (siSOX2 *vs* cntr). γ-TUB was used as a normalization control for protein quantification. **(B-C)** Uncropped plot relative to Western Blot analyses (showed in Fig 4C) of EMT markers (E-CAD, VIM, SLUG, SNAIL) in CAL27 and SCC15 cells (siSOX2 *vs* cntr). γ-TUB was used as a normalization control for protein quantification. **(D)** Uncropped plot relative to Western Blot analyses (showed in Fig 4D) of AKT and p-AKT in CAL27 and SCC15 cells (siSOX2 *vs* cntr). γ-TUB was used as a normalization control for protein quantification. **(E)** Uncropped blot relative to Western Blot analysis (showed in S1 Fig) of SOX2 in T, CM and DM samples of two representative patients (#1 and #2). RPL38 was used as a normalization control for protein quantification.
(DOCX)

S1 Movie Time lapse of wound healing assay of CAL27 cntr for 72h (10x magnification). (MP4)

S2 Movie Time lapse of wound healing assay of CAL27 siSOX2 for 72h (10x magnification). (MP4)

S3 Movie Time lapse of wound healing assay of SCC15 cntr for 72h (10x magnification). (MP4)

S4 Movie Time lapse of wound healing assay of SCC15 siSOX2 for 72h (10x magnification). (MP4)

S5 Movie Time lapse of 3D tumor spheroid formation assay of CAL27 cntr for 6 days (10x magnification). (MP4)

S6 Movie Time lapse of 3D tumor spheroid formation assay of CAL27 siSOX2 for 6 days (10x magnification). (MP4)

S7 Movie Time lapse of 3D tumor spheroid formation assay of SCC15 cntr for 6 days (10x magnification). (MP4)

S8 Movie Time lapse of 3D tumor spheroid formation assay of SCC15 siSOX2 for 6 days (10x magnification). (MP4)
