# Supplementary material for: SOX2 promotes a cancer stem cell-like phenotype and local spreading in oral squamous cell carcinoma
Source: PLoS One. 2023 Dec 14;18(12):e0293475. doi: 10.1371/journal.pone.0293475 (PMC10721099; doi:10.1371/journal.pone.0293475)
Supplement: S1 Fig — WB analysis of SOX2 in T, CM, and DM samples of two representative OSCC patients (#1 and #2) and relative optical densitometry. In patient #1, SOX2 protein level is higher in T compared to CM and DM. In patient #2, SOX2 protein level is similar in T and CM and higher compared to DM. RPL38 was used as a normalization control for protein quantification. (PDF) [file pone.0293475.s001.pdf]

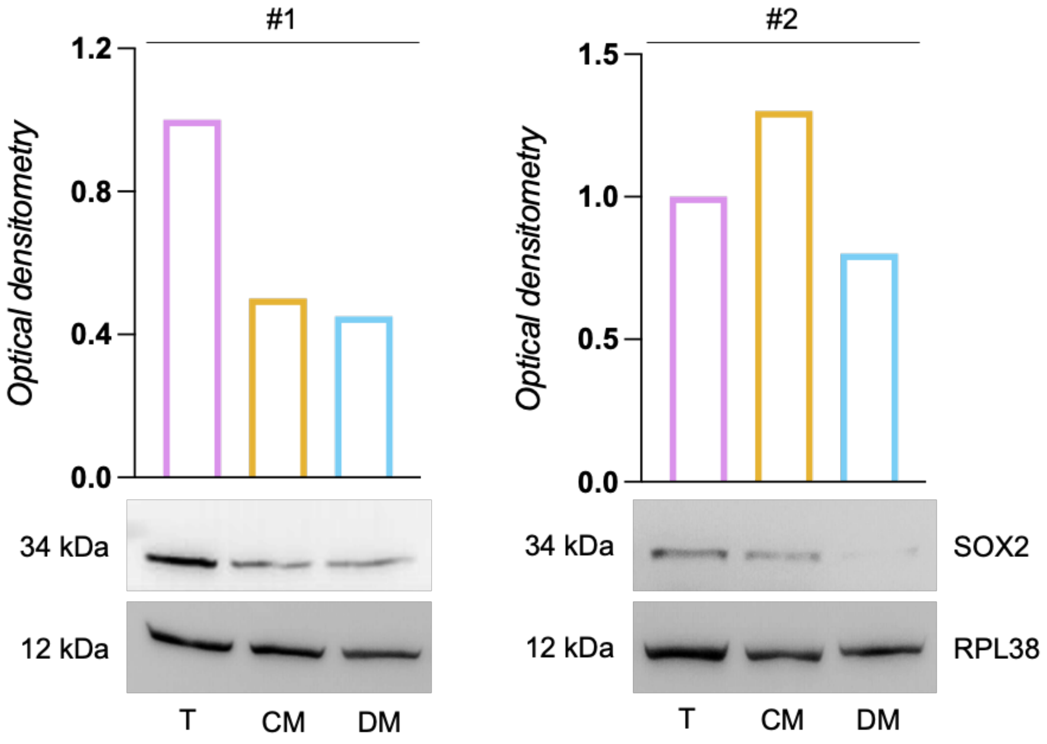

S1 Fig. SOX2 protein levels in T, CM, and DM of two representative OSCC samples. WB analysis of SOX2 in T, CM and DM samples of two representative OSCC patients (#1 and #2) and relative optical densitometry. In patient #1, SOX2 protein level is higher in T compared to CM and DM. In patient #2, SOX2 protein level is similar in T and CM and higher compared to DM. RPL38 was used as a normalization control for protein quantification.
