# Supplementary material for: SOX2 promotes a cancer stem cell-like phenotype and local spreading in oral squamous cell carcinoma
Source: PLoS One. 2023 Dec 14;18(12):e0293475. doi: 10.1371/journal.pone.0293475 (PMC10721099; doi:10.1371/journal.pone.0293475)
Supplement: S2 Fig — Box plots showing NANOG and RRM2 gene expression levels (log2 |FC|) in T, CM, and DM samples (ns: not significant). (PDF) [file pone.0293475.s002.pdf]

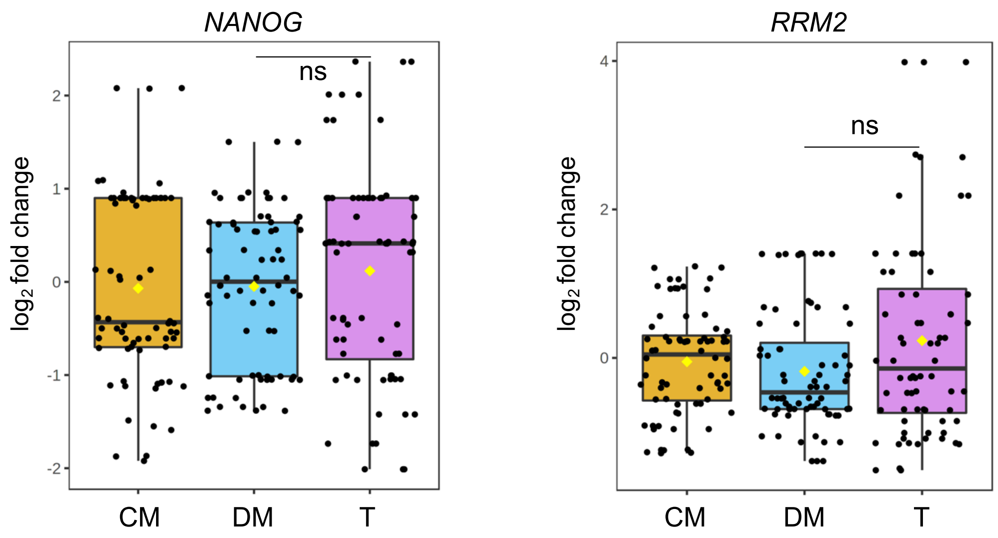

S2 Fig. *NANOG* and *RRM2* did not show any significant difference among T, CM and DM samples. Box plots showing *NANOG* and *RRM2* gene expression levels ($\log_2$ |FC|) in T, CM, and DM samples (ns: not significant).
