# Supplementary material for: SOX2 promotes a cancer stem cell-like phenotype and local spreading in oral squamous cell carcinoma
Source: PLoS One. 2023 Dec 14;18(12):e0293475. doi: 10.1371/journal.pone.0293475 (PMC10721099; doi:10.1371/journal.pone.0293475)
Supplement: S3 Fig — Box plots showing the relative expression of VIM and E-CAD in both T (A) and CM (B) samples clustered according to high or low SOX2 levels, respectively (*ANOVA t-test p value < 0.05). (PDF) [file pone.0293475.s003.pdf]

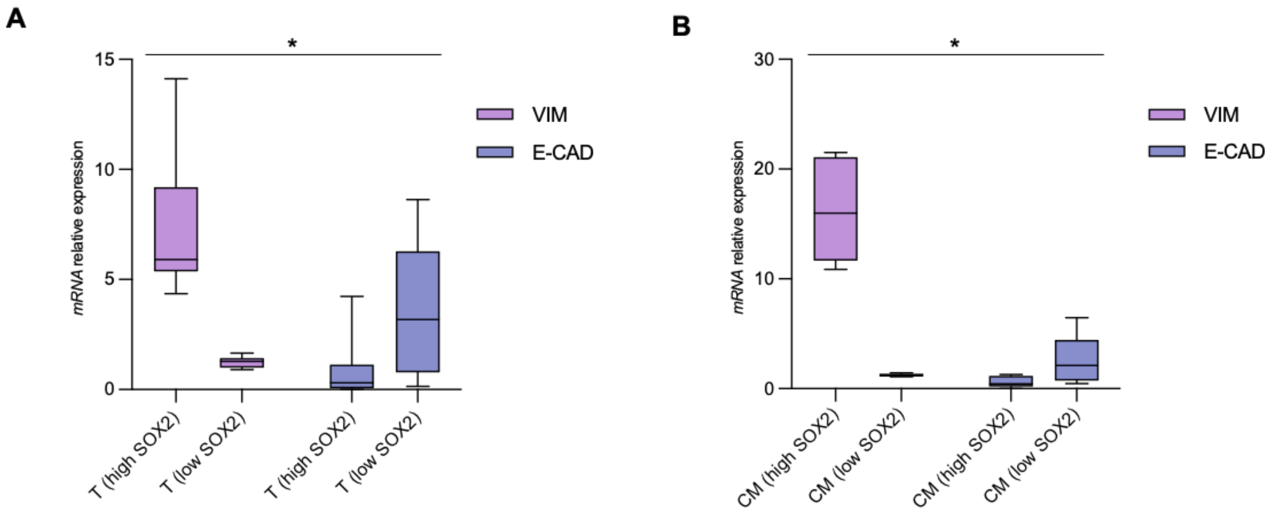

S3 Fig. High *SOX2* levels correlate with EMT phenotype in tissue samples. Box plots showing the relative expression of *VIM* and *E-CAD* in both T **(A)** and CM **(B)** samples clustered according to high or low *SOX2* levels, respectively (*ANOVA t-test p value < 0.05).
