# Supplementary material for: SOX2 promotes a cancer stem cell-like phenotype and local spreading in oral squamous cell carcinoma
Source: PLoS One. 2023 Dec 14;18(12):e0293475. doi: 10.1371/journal.pone.0293475 (PMC10721099; doi:10.1371/journal.pone.0293475)
Supplement: S1 Table — (PDF) [file pone.0293475.s004.pdf]

S1 Table. Details of the selected four databases.

EXPERIMENT ID	DOI	SAMPLES	LESION SITE
GSE64216	-	4 (2 OSCC + 2 non-OSCC)	Oral buccal mucosa
GSE138206	-	18 (6 OSCC + 6 adjacent tissues + 6 non-OSCC)	15 tongues + 3 oral buccal mucosae
GSE74530	10.1002/ijc.30177	12 (6 OSCC + 6 adjacent non OSCC)	Oral epithelia
GSE37991	10.1002/path.4173	80 (40 OSCC + 40 non-OSCC)	Oral epithelia
