# Supplementary material for: SOX2 promotes a cancer stem cell-like phenotype and local spreading in oral squamous cell carcinoma
Source: PLoS One. 2023 Dec 14;18(12):e0293475. doi: 10.1371/journal.pone.0293475 (PMC10721099; doi:10.1371/journal.pone.0293475)
Supplement: S1 Raw images — (A) Uncropped plot relative to Western Blot analysis (showed in Fig 4A) of SOX2 in CAL27 and SCC15 cells (siSOX2 vs cntr). γ-TUB was used as a normalization control for protein quantification. (B-C) Uncropped plot relative to Western Blot analyses (showed in Fig 4C) of EMT markers (E-CAD, VIM, SLUG, SNAIL) in CAL27 and SCC15 cells (siSOX2 vs cntr). γ-TUB was used as a normalization control for protein quantification. (D) Uncropped plot relative to Western Blot analyses (showed in Fig 4D) of AKT and p-AKT in CAL27 and SCC15 cells (siSOX2 vs cntr). γ-TUB was used as a normalization control for protein quantification. (E) Uncropped blot relative to Western Blot analysis (showed in S1 Fig) of SOX2 in T, CM and DM samples of two representative patients (#1 and #2). RPL38 was used as a normalization control for protein quantification. (DOCX) [file pone.0293475.s005.docx]

**S1_raw_images**

**B**

**A**

**
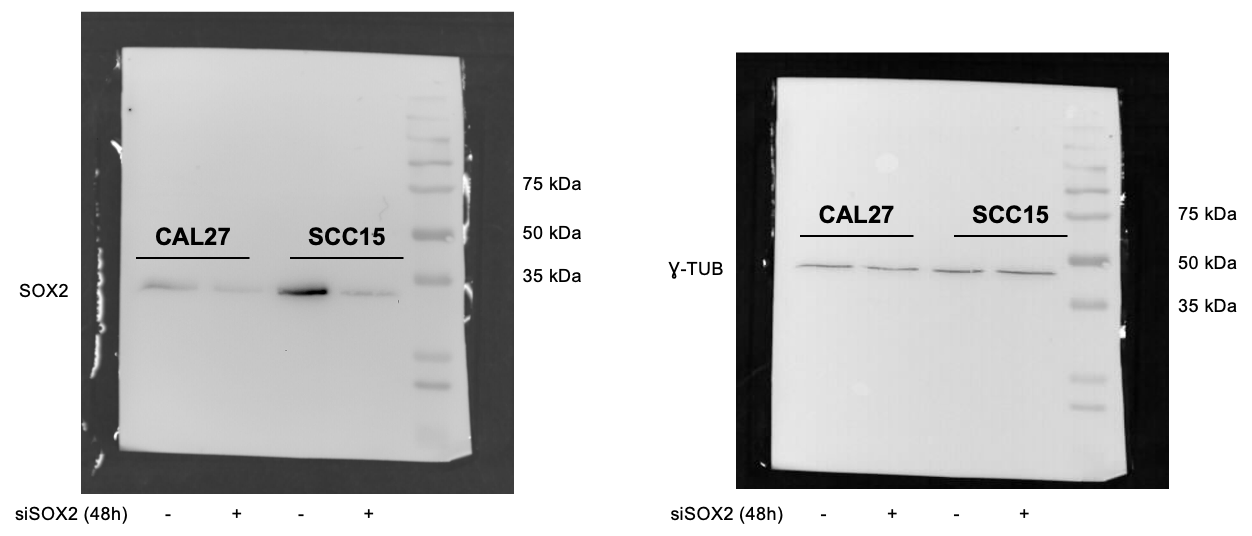
**

**
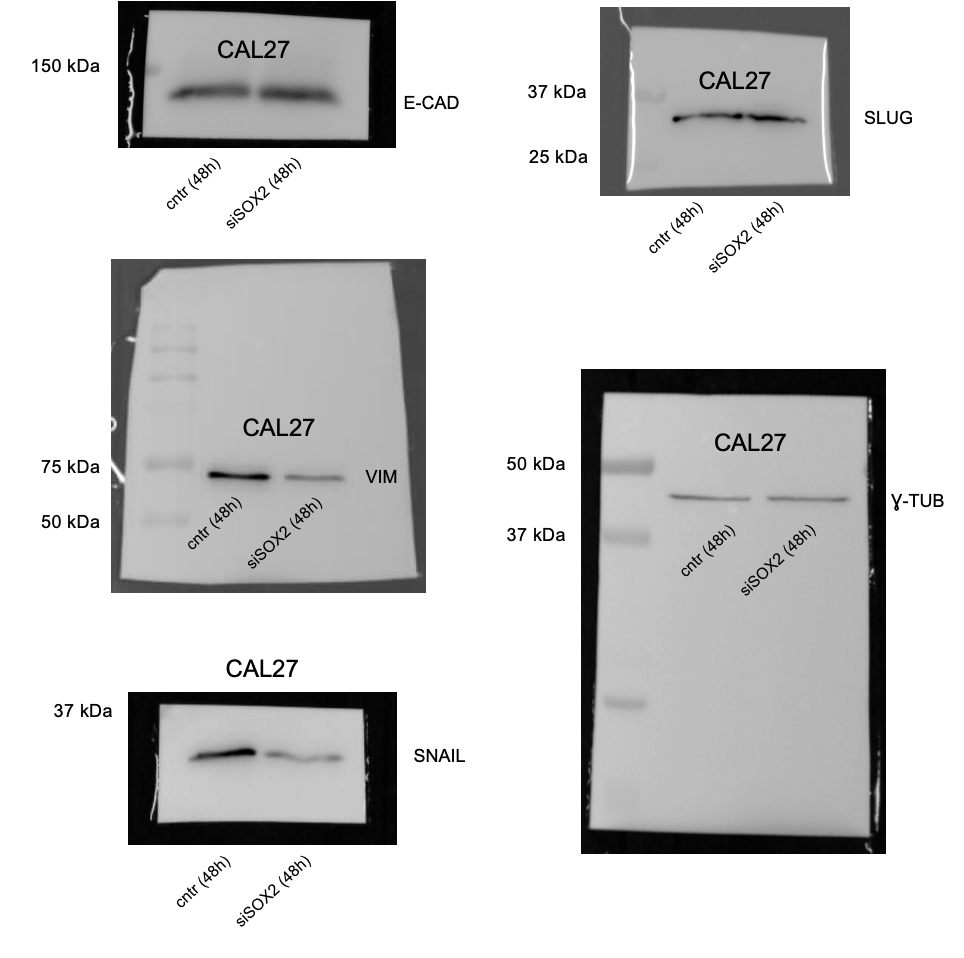
**

**
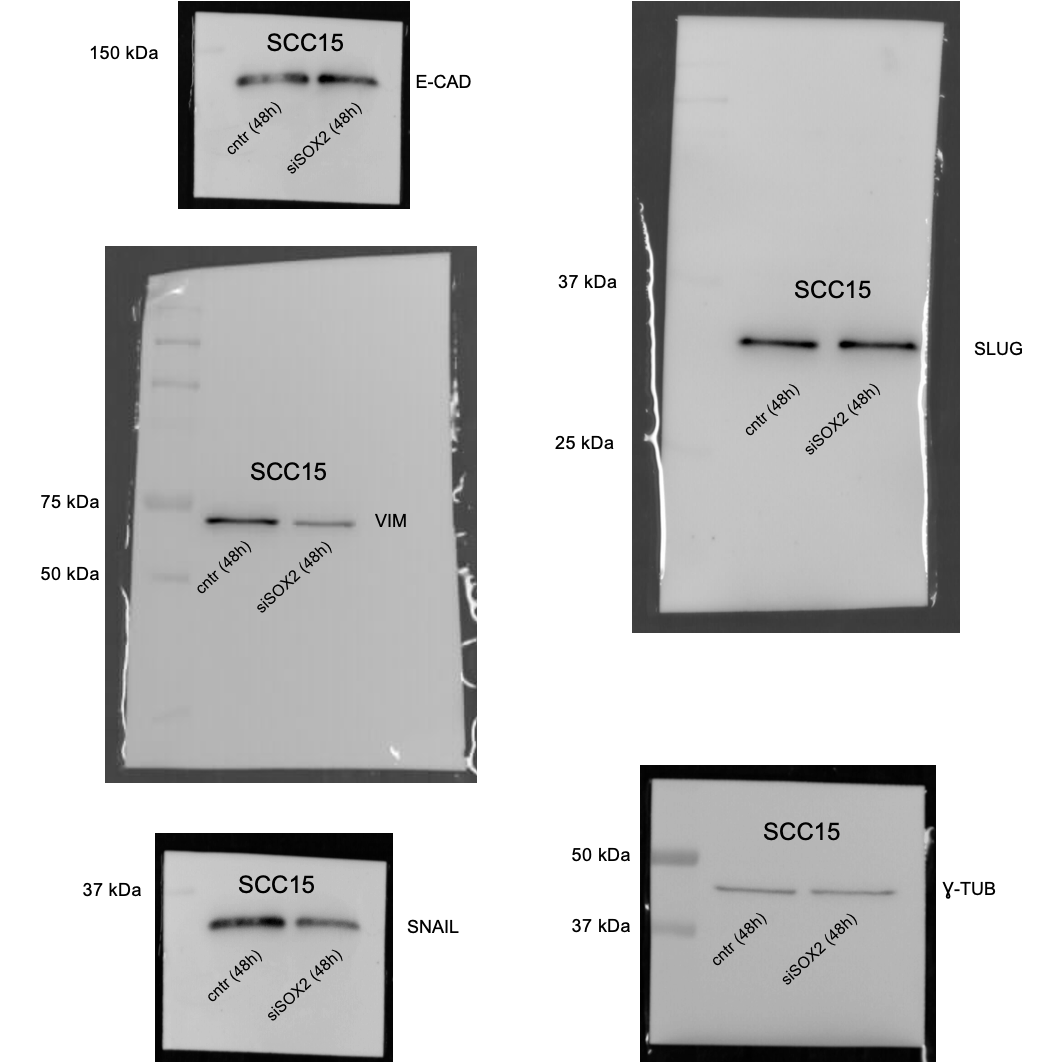
**

**C**

**D**

**
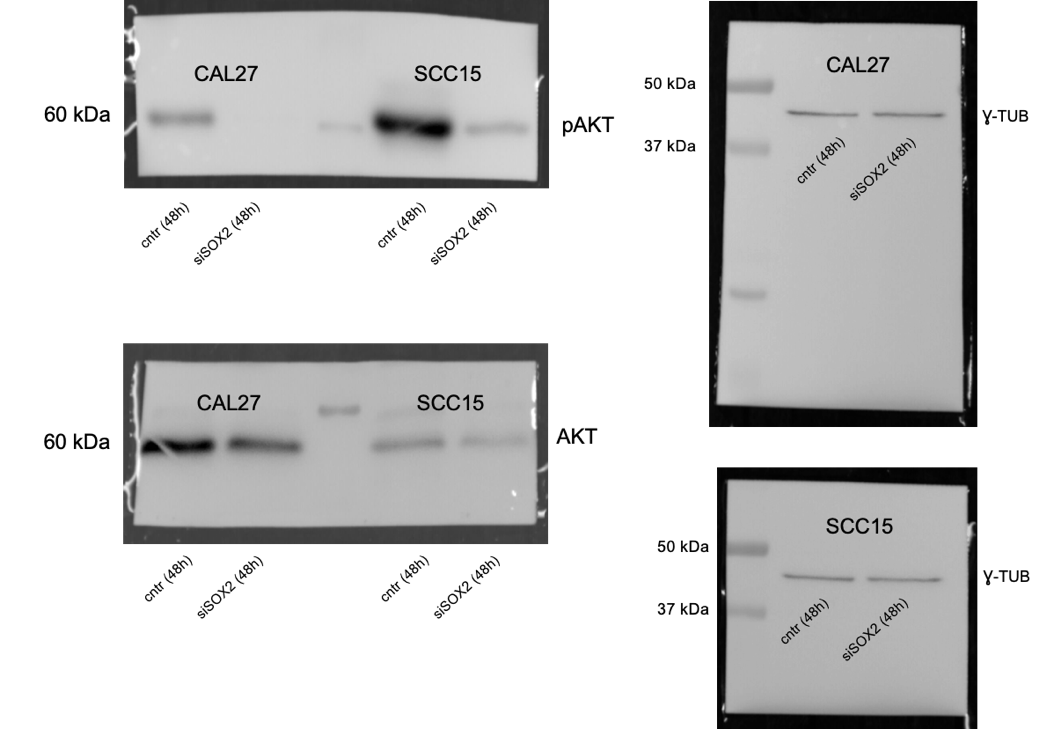
**

**
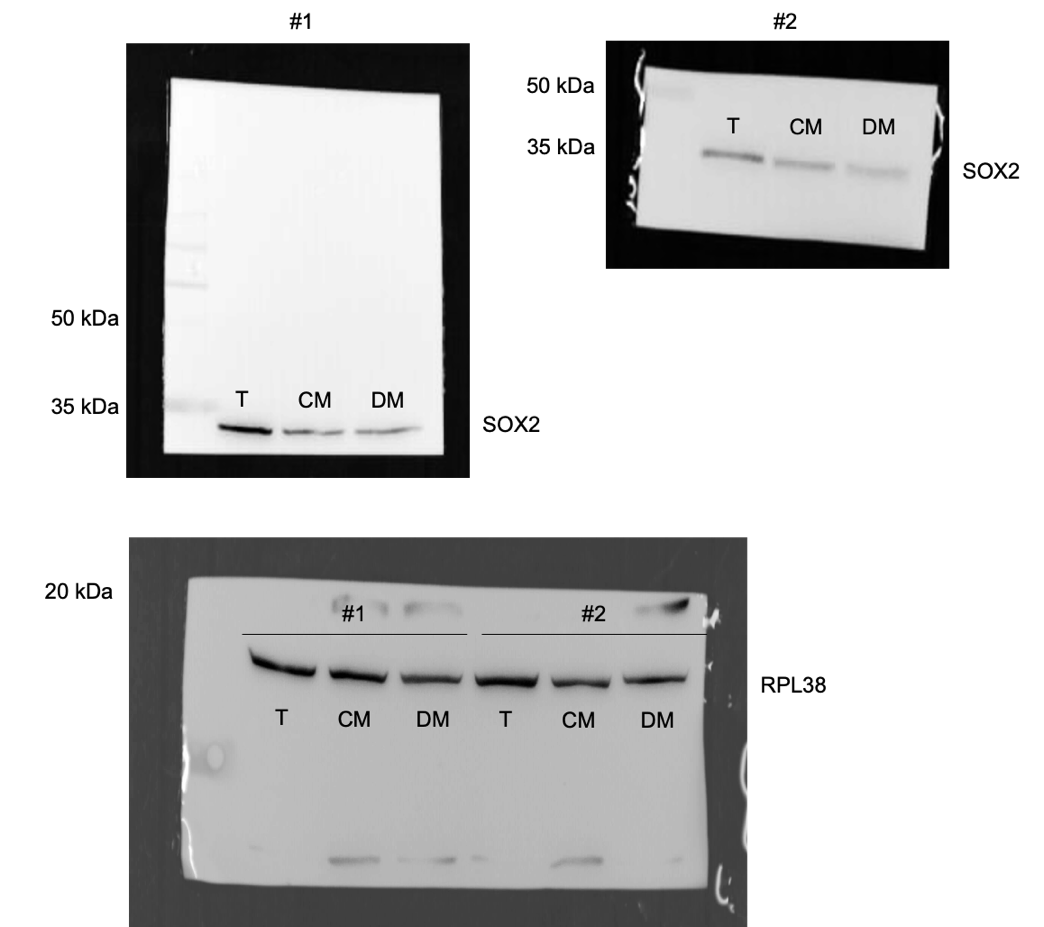
**

**E**

**S1_raw_images.** **Uncropped plots relative to all Western Blot analyses. (A)** Uncropped plot relative to Western Blot analysis (showed in Fig 4A) of SOX2 in CAL27 and SCC15 cells (siSOX2 *vs* cntr). γ-TUB was used as a normalization control for protein quantification. **(B-C)** Uncropped plot relative to Western Blot analyses (showed in Fig 4C) of EMT markers (E-CAD, VIM, SLUG, SNAIL) in CAL27 and SCC15 cells (siSOX2 *vs* cntr). γ-TUB was used as a normalization control for protein quantification. **(D)** Uncropped plot relative to Western Blot analyses (showed in Fig 4D) of AKT and p-AKT in CAL27 and SCC15 cells (siSOX2 *vs* cntr). γ-TUB was used as a normalization control for protein quantification. **(E)** Uncropped blot relative to Western Blot analysis (showed in S1 Fig) of SOX2 in T, CM and DM samples of two representative patients (#1 and #2). RPL38 was used as a normalization control for protein quantification.
